# Old and Cold: A Novel Case of Combined Secobarbital and Pentobarbital Poisoning in an Elderly Woman

**DOI:** 10.7759/cureus.12446

**Published:** 2021-01-03

**Authors:** Kenneth D Katz, Andrew Koons, Gregory Makar, Amy Wier

**Affiliations:** 1 Emergency Medicine • Medical Toxicology, University of South Florida Morsani College of Medicine / Lehigh Valley Health Network, Allentown, USA; 2 Emergency Medicine, University of South Florida Morsani College of Medicine / Lehigh Valley Health Network, Allentown, USA

**Keywords:** barbiturate overdose, secobarbital poisoning, pentobarbital poisoning, elderly patient, hypothermia

## Abstract

A 94-year-old woman was found unresponsive in her room at an independent living facility. Upon paramedic arrival, the patient had a Glasgow Coma Scale of 3, and she was transported to the emergency department (ED). In the ED, she was unresponsive but spontaneously breathing, bradycardia, and hypothermia. Serum concentrations of both secobarbital (3.3µg/mL; therapeutic 1.0-2.0µg/mL) and pentobarbital (9.5µg/mL; therapeutic < 5.0µg/mL) were detected and elevated. This type of poisoning is quite rare and should be considered in patients presenting with hypothermia and coma, even in patients showing brain death signs. The use of hemodialysis for refractory pentobarbital poisoning may be helpful.

## Introduction

Barbiturates are sedative medications that agonize the gamma-aminobutyric acid receptor in the central nervous system (CNS), causing the chloride channel's prolonged opening. The neuronal hyperpolarization effect is decreased neuronal firing and CNS depression [1]. Commonly prescribed barbiturates include phenobarbital, which is used for epilepsy and alcohol withdrawal, and butalbital, which is used for epilepsy and headache [1]. Additionally, pentobarbital is used as a general anaesthetic in both humans and animals [2]. Secobarbital is prescribed only as a primary euthanasia agent where physician-assisted suicide has been legalized [2]. Though diagnosing barbiturate toxicity can be difficult, it is essential to recognize some of its potential effects such as severely depressed mental status and hypothermia. An extraordinarily unique case of an elderly patient suffering from a combined overdose of both secobarbital and pentobarbital is described.

## Case presentation

A 94-year-old woman with a past medical history significant for Non-Hodgkin's splenic marginal zone lymphoma, hemolytic anemia, hepatocellular carcinoma, hypertension, neurogenic bladder, osteoarthritis, and shingles prescribed aspirin, atenolol, cholecalciferol, and losartan was found obtunded in her room at an independent living facility. Upon paramedic arrival, she was breathing spontaneously and maintaining adequate oxygenation, even though her Glasgow Coma Scale was 3. She was transported to the emergency department (ED) for further evaluation. 

In the ED, the patient was responsive to noxious stimuli only but was spontaneously breathing. Vital signs included: heart rate 56 beats per minute, blood pressure 111/54mm of mercury (mm Hg), temperature 35.7ºC (temporal), respirations 16 breaths per minute, and a room air pulse oximetry saturation of 96%. Her physical examination was otherwise unremarkable. Fingerstick glucose measured 106 mg/dL(normal: 65-99 mg/dL). Initial routine laboratory (including serum acetaminophen, salicylate and ethanol concentrations) and radiographic testing- computed tomography head, chest, abdomen, and pelvis - was unremarkable. A rapid urine drug screen of abuse preliminarily detected barbiturates. In the ED, the patient's family arrived and expressed concerns regarding pills found in the patient's room, which had not been prescribed. These pills, however, were not able to be identified. She was subsequently admitted to the intensive care unit for further monitoring.

The patient's vital signs normalized, mental status gradually improved, and her coma fully resolved by hospital day (HD) 3. She underwent extensive neurologic testing during this time, including magnetic resonance imaging of the brain, showing small remote vessel ischemic disease and an electroencephalogram that demonstrated diffuse background slowing but no seizures. She did not receive hemodialysis because her living will forbid it. Serum liquid chromatography/mass spectroscopy toxicology testing on blood drawn upon ED presentation detected both secobarbital and pentobarbital. Initial serum concentrations measured elevated levels of both secobarbital at 3.3µg/mL (therapeutic 1.0-2.0µg/mL, toxic >3.0µg/mL) and pentobarbital at 9.5µg/mL (hypnotic 1.0-5.0µg/mL, toxic >10.0µg/mL), respectively [3,4]. On HD 3, the patient was transferred to the medical floor in stable condition. The patient denied any knowledge of either drug or the events of her hospitalization. She adamantly denied an overdose and stated she did not have access to these medications. Repeat serum testing on HD 5 detected no secobarbital and pentobarbital. The patient ultimately improved and was discharged to a skilled nursing facility on HD 11.

## Discussion

Barbiturates are infrequently encountered in the outpatient setting due to increased use of benzodiazepines, which exhibit a wider therapeutic window and lower incidence of single-substance fatal overdose. Barbiturate poisoning can be especially difficult to diagnose in the elderly, who have a higher incidence of organic etiologies of altered mental status. Previous research has demonstrated patient age to be highly predictive of severe intoxication [5]. This may be due to slower hepatic elimination and decreased physiologic compensation capability in elderly patients. Moreover, the duration of clinically significant signs and symptoms after barbiturate overdose correlates poorly with pharmacokinetic half-life [6]. Case reports have described brain stem reflexes' return even five days after initial examination showed evidence of brain death [5]. Historically, pentobarbital has been used in veterinary euthanasia medicine. However, case reports of fatal exposure after obtaining the medication from China and Mexico have been published within the last five years [7,8]. Secobarbital is reportedly the most common medication used in physician-assisted suicide [9]. However, accidental intoxication with secobarbital has rarely been reported. Based on a review of available literature, this is a novel case of combined secobarbital and pentobarbital intoxication in a single patient.

Treatment of barbiturate poisoning is predominantly supportive. Hemodialysis for severe hypotension and refractory coma after pentobarbital poisoning has been described with variable efficacy [10]. Unlike phenobarbital, which has shown increased elimination with urinary alkalization, secobarbital and pentobarbital have higher pKa values that do not allow for effective excretion of the un-metabolized parent drug. Historically, enhanced elimination with multi-dose activated charcoal has been used in phenobarbital overdoses with improved clearance, but without clear evidence of improvement in clinical outcome [11].

## Conclusions

This is a unique case of an elderly patient suffering from a combined overdose of both secobarbital and pentobarbital. Barbiturate toxicity should be considered in patients presenting with hypothermia and coma, even in those exhibiting apparent clinical signs of brain death. Due to the elderly having higher chances of organic etiologies of alerted mental status, barbiturate poisoning can be especially challenging to diagnose in this age group. Additionally, the use of hemodialysis may be helpful for refractory pentobarbital poisoning.
